# Synthesis and characterization of nano bioactive glass for improving enamel remineralization ability of casein phosphopeptide–amorphous calcium phosphate (CPP-ACP)

**DOI:** 10.1186/s12903-022-02549-9

**Published:** 2022-11-24

**Authors:** Tina Akbarzade, Abbas Farmany, Maryam Farhadian, Zahra Khamverdi, Ramtin Dastgir

**Affiliations:** 1 Department of Restorative Dentistry, School of Dentistry, Hormozgan University of Medical Sciences, Bandar-Abbas, Iran; 2 Dental Research Center, Dental Implant Research Center, School of Dentistry, Hamadan University of Medical Sciences, Hamadan, Iran; 3 School of Public Health, Hamadan University of Medical Sciences, Hamadan, Iran; 4 Department of Operative Dentistry, School of Dentistry, Hamadan University of Medical Sciences, Hamadan, Iran; 5 Faculty of Dentistry, Tehran Branch, Islamic Azad University, Tehran, Iran

**Keywords:** Nano bioactive glass, Tooth remineralization, Casein phosphopeptide-amorphous calcium phosphate, Microhardness

## Abstract

**Objective:**

Nanomaterials with superior properties such as high surface area over volume ratio are widely used in dentistry and medicine. This in vitro study was performed to synthesize and characterize nano bioactive glass (nBG) and to evaluate the effect of casein phosphopeptide-amorphous calcium phosphate (CPP-ACP) containing nBG (CPP-ACP@nBG) on enamel remineralization by its application to pH-cycled, synthetically demineralized enamel surfaces.

**Materials and methods:**

nBG particles were prepared by sol-gel method. X-ray diffraction pattern (XRD), Fourier-transform infrared spectroscopy (FTIR) and transmittance electron microscopy (TEM) were used for nBG characterization. Synthetic CPP-ACP paste was prepared and nBG particles were added to it. To evaluate the degree of remineralization, 32 healthy human premolars were selected. The samples were randomly divided into 4 groups as: Group 1: Commercial CPP-ACFP (MI paste plus), Group 2: Synthetic casein phosphopeptide-amorphous calcium phosphate containing fluoride (CPP-ACP@F), Group 3: Synthetic CPP/ACP containing nBG (CPP-ACP@nBG), and Group 4: Control (received no treatment). The pastes were then applied on the tooth surfaces for 28 days. The Vickers microhardness of enamel surfaces was evaluated, and enamel surface morphology was assessed using scanning electron microscopy (SEM).

**Results:**

X-Ray diffraction pattern (XRD) of the synthesized nBG show its crystalline nature with the Larnite crystalline mode. Transmittance electron microscope (TEM) microimage of the synthesized nBG shows its formation as less that 100 nm spherical nanoparticle with partial agglomeration. Fourier transform infrared spectroscopy (FTIR) confirm the success formation of nBG with high purity. The results of this study showed that microhardness of the experimental groups was significantly higher than the control group (p ≥ 0.05). SEM images showed a layer of hydroxyapatite in the CPP-ACP@nBG, synthetic and commercial CPP-ACP@F remineralized groups.

**Conclusion:**

The results of this study demonstrated that CPP-ACP@F and CPP-ACP@nBG remineralize the surface of the demineralized enamel. Microhardness of the remineralized enamel in the CPP-ACP@nBG group was higher than synthetic and commercial CPP-ACP@F groups.

## Introduction

In the oral environment, the processes of demineralization and remineralization are in balance, however, some conditions such as unhealthy diet, lack of oral hygiene and dental caries can change this balance and dominate the demineralization process [1, 2]. Cariogenic bacteria accumulate on the enamel biofilm surface, in the presence of fermentable carbohydrates and form organic acids such as lactic acid which initiate the demineralization process by decreasing the pH [1]. In recent decades, with spread of “minimally invasive dentistry” approach and its emphasis on prevention, enamel areas with caries are treated by preventing the demineralization process and improving the remineralization conditions without removing excessive amounts of tooth tissue [2–4].

Casein phosphopeptide amorphous calcium phosphate (CPP-ACP) was used for the enamel remineralization. CPP is a multi-phosphorylated peptide derived from casein and binds to calcium and phosphate ions through phosphoryl residues and stabilize them as ACP [5, 6]. This compound binds to the tooth surface via CPP and causes the release of calcium, phosphate and fluoride by super saturation of these ions on the tooth surface. Furthermore, CPP-ACP can bind to the pellicle, plaque, soft tissue, and enamel hydroxyapatite [3, 5]. It was shown that CPP-ACFP, which contains fluoride (0.09%), is more effective than CPP-ACP in the enamel remineralization process [2, 3, 7]. Fluorapatite has the potential to increase the calcium and phosphate deposition, which in turn balances the remineralization and demineralization process; however, this does not completely eliminate the risk of caries formation [5, 6]. On the other hand, bioactive substances such as bioactive glass, calcium phosphate, hydroxyapatite, and calcium silicate can repair and regenerate the tooth enamel [8]. Bioactive glass has been widely used in the enamel remineralization due to its similar structure to bone and enamel [9]. The release of sodium, calcium, and phosphate ions from bioactive glass, is followed by the formation of carbonated calcium-deficient hydroxyapatite (HCA). This component is used as a suitable candidate for enamel remineralization and is widely used in toothpastes, air polishing, and treatment of dentin hypersensitivity [6, 10, 11].

Numerous in vitro and in vivo studies have demonstrated that bioactive glasses do not usually lead to microleakage in contact with living cells and does not cause tissue inflammation. It is a biocompatible substance without any adverse effect on the pulp cells in the treatment of enamel and dentin sensitivity [4]. Furthermore, bioactive glass is more effective in remineralization of enamel than other topical remineralizing agents [12].

Today, nanomaterials, are widely used in medicine and dentistry due to superior properties such as high surface area over volume ratio etc.… [13]. Because of the positive effects of CPP-ACP and the desirable properties of bioactive glass in the tooth remineralization process, this in vitro study was designed to synthesize CPP-ACP paste containing nano bioactive glass (nBG) and to evaluate its effect on the demineralized enamel surfaces using Vickers microhardness test and scanning electronic microscope (SEM).

## Materials and methods

### Synthesis of nano bioactive glass

Nano bioactive glass (nBG) was synthesized using a modified sol-gel method [14, 15]. Briefly, 16.8 mL tetraethyl orthosilicate (TEOS) was mixed with calcium nitrate in a water/ethanol (2:1) solution. The pH of solution was adjusted to 2 by addition of citric acid (1 M). Stirring was continued until a clear solution (solution A) was obtained. A solution of 2% polyethylene glycol (PEG) (MW: 2000) and diamonium hydrogen orthophosphate was prepared and by addition of ammonia, its pH was adjusted to 10 (solution B). Solutions A and B were mixed under stirring for 10 h to obtain a homogeneous gel. It was washed and filtered with deionized double distilled water. The obtained white gel was dried and lyophilized using an electrical oven at 60 °C for 8 h, and finally calcined for 10 h at 650 °C.

### Synthesis of CPP-ACP paste

A solution of casein (10% w/v) was prepared at pH 8 (pH was adjusted with NaOH) and then an appropriate amount of trypsin (0.2 w/w relative to casein) was dissolved in the solution and stirred for 2 h at 50 °C. The pH of solution was adjusted to 4.6 by addition of HCl. It was centrifuged and the pH of the obtained precipitate was adjusted to 9 by addition of NaOH. Then, CaCl_2_ (1 mol), Na_2_HPO_4_ (1 mol) and NaF (200 mmol) were slowly added to the solution during continuous shaking to obtain the final concentration of calcium chloride (100 mM Ca (II)), sodium phosphate (60 mM phosphate) and sodium fluoride (12 mM fluoride) [16, 17]. The solution was filtered using a microfilter (0.1–0.2 μm) and the obtained precipitate was spray dried. The obtained white powder containes 50% CPP and 40% ACP [16, 17]. Finally, CPP-ACP@nBG and CPP-ACP@F were prepared by addition of 0.5% w/w nBG or sodium flouride to the CPP-ACP paste.

### Characterization of nano bioactive glass

X-Ray diffraction pattern (XRD) was used to investigate the crystal structure of nBG. Panalytical Xpert PRO X Ray Diffractometer (Panalytical, Netherlands) model Xpert Pro MPD with wavelength 1.5405 Å and power 40KV/30mA was used to study the structure and crystal phase of the nBG by scanning in the range of 15 to 80 degrees.

Fourier transform infrared spectroscopy (FTIR) was used to assess the functional groups and chemical structure and bonds of the synthesized nBG. FTIR spectra of the synthesized nBG were recorded by an FT-IR Spectrometer (PerkinElmer, USA) model Spectrum400 in the frequency range of 400–4000 cm^− 1^. The morphology, size distribution and shape of nBG particles were assessed by transmittance electron microscope (TEM) model Philips XL30 ESEM (Netherlands).

### Sample preparation

The study protocol was approved by the Ethics Committee of Hamadan University of Medical Sciences (No# IR.UMSHA.REC.1398.843). Premolars that had been extracted due to orthodontic or prosthetic treatment, were collected and kept in chloramine (0.5%). A written consent form was obtained from all patients over the age of 16 and from legal guardians of patients under the age of 16 prior to extraction of their teeth. A sharp blade was used to clean soft tissue and calculus around the extracted teeth and were subsequently polished. The premolars were examined by stereo microscope (Olympus, Shinjuku, Tokyo, Japan) and 32 premolars without cracks or lesions were selected. The crown of the teeth was removed from the CEJ area by a diamond saw (Micro slice 2, Metal Research, Cambridge, UK) and then the crowns were cut into facial and palatal halves. Each half was mounted in acrylic resin (Acropars, Kaveh, Tehran, Iran) exposing 4 x 4 mm of enamel surface. Polishing procedure incorporated on the exposed enamel, by using of 600-grit, 800-grit, 1200-grit silicon carbide papers subsequently and were thoroughly rinsed with deionized double distilled water after each gritting. The mounted samples were placed in deionized double distilled water to prevent the dehydration.

For demineralization, all mounted samples were immersed in the demineralizing solution (2.2 mM CaCl_2_ • 2H_2_O, 2.2 mM KH_2_PO_4_, 50 mM sodium acetate) for 96 h at 37 °C and pH of 4.4 [18].

After washing with deionized double distilled water, the samples were pH cycled in demineralization (2.2 mM CaCl_2_ • 2H_2_O, 2.2 mM KH_2_PO_4_, 50 mM sodium acetate) and remineralization (20 mM 4-(2-hydroxyethyl) -1 piperazineethanesulfonic acid (HEPES), 1.5 mM CaCl_2_, 0.9 mM KH_2_PO_4_, 150 mM KCl) solutions. The pH cycling procedure constituted cycling in the demineralizing solution at pH of 4.4 for 30 min and then for 10 min in remineralizing solution at pH of 7.0 at room temperature. This cycle was repeated 6 times a day for 8 days and the samples were kept in deionized double distilled water during the night [18]. The samples were randomly divided into 4 groups as follows. G1: commercial CPP-ACF@P (MI paste plus); G2: synthetic CPP-ACP@F; G3: synthetic CPP-ACP@nBG and G4: control, in which demineralization process was conducted, however, no reminrelization process was undertaken.

In the experimental groups, the paste (0.1 g of material was mixed with 3 mL of deionized double distilled water for 1 min to form a paste) was applied directly on the surface of the demineralized enamel using a micro-brush for 4 min (twice a day at 8 am and 4 pm for 28 days) at room temperature. Then, the samples were washed using a micro-brush and deionized double distilled water and kept in deionized double distilled water for 48 h at room temperature [19].

To evaluate the remineralization of enamel surfaces and measure the surface microhardness, a Vickers microhardness test was used. Microhardness was measured at 3 points on the surface of each sample, and the mean microhardness was calculated. Indentation was generated with a force of 500 g for 5 s and was calculated by the use of a microhardness tester microscope (Micrometer 1, Buehler, Lake Bluff, IL, USA).

A scanning electronic microscope (Hitachi S-450, 20 kV, Japan) was used to assess the surface morphology of enamel samples. Samples were dehydrated using ethanol and were subsequently coated with a layer of gold prior to analysis.

### Statistical analysis

Data were analyzed using SPSS version 21 software (IBM Corp., Armonk, NY, USA). Kolmogorov-Smirnov test was used to evaluate the normality of microhardness data distribution. Due to the normality of data, one-way analysis of variance (ANOVA) and Tukey post hoc tests were used for comparison between the studied groups. The confidence level was set as 95% (α = 0.05).

### Sample size

The minimum sample size required in this study was determined as 16 (64 samples in 4 groups) [18, 20]. The reliability of the test was 95% and the test power was 80%. The expected difference was considered in the average of $\mu_1-\mu_2=30$ and the standard deviation was $\sigma=30$.

## Results

### Nano bioactive glass characterization

**Fig. 1:**
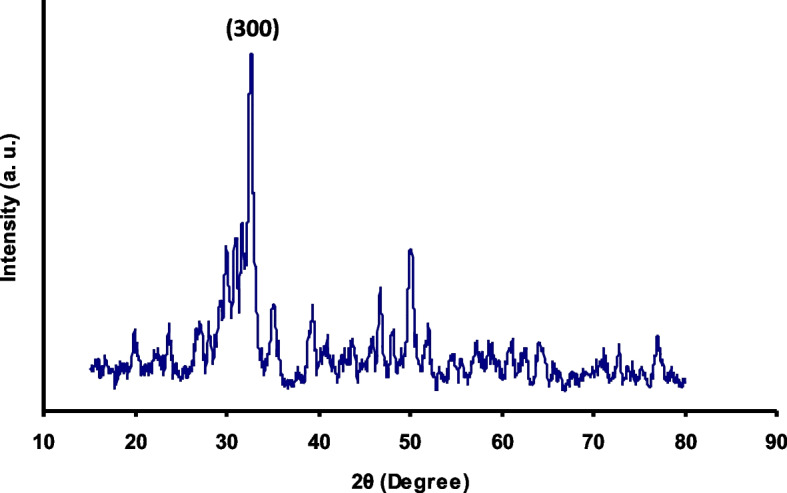
X-Ray Diffraction (XRD) pattern of nBG

**Fig. 2:**
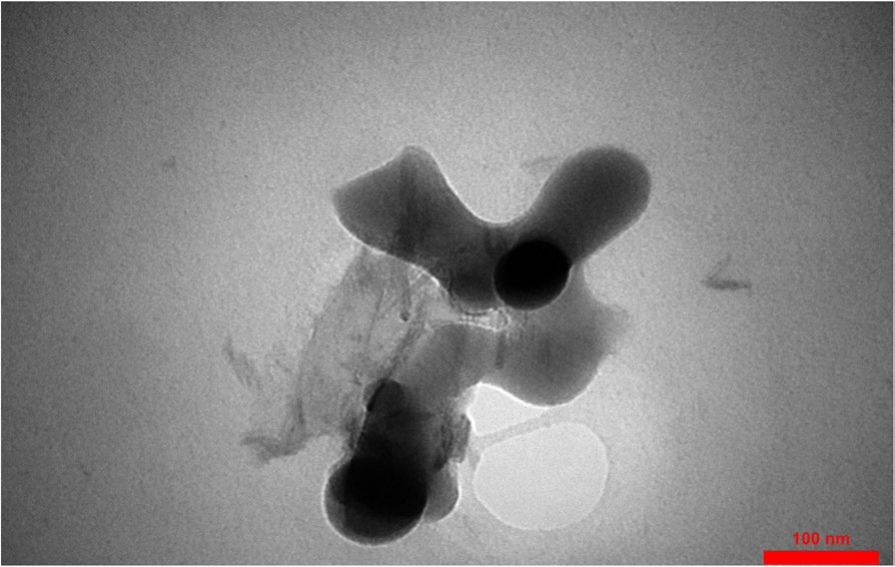
Transmission electron microscopy (TEM) image of nBG

X-Ray diffraction (XRD) profile of the synthesized nBG is shown in Fig. 1. The XRD peaks of calcined nanoparticles at 650 °C are consistent with the Larnite crystalline mode corresponding to Ca_2_SiO_4_ (JCPDS # 33–0302) [21]. A sharp peak shown in 2θ = 32.08 corresponds to the Miller index (300). The presence of a sharp peak in this area indicates the high crystallization of the synthesized nanoparticles. Transmittance electron microscope (TEM) image of the synthesized nBG shows partial agglomeration of nanoparticles (Fig. 2) [21, 22]. As shown in TEM image of synthesized nanoparticles, their size is less than 100 nm, which is necessary to obtain superior biological activity compared to large crystals.

**Fig. 3:**
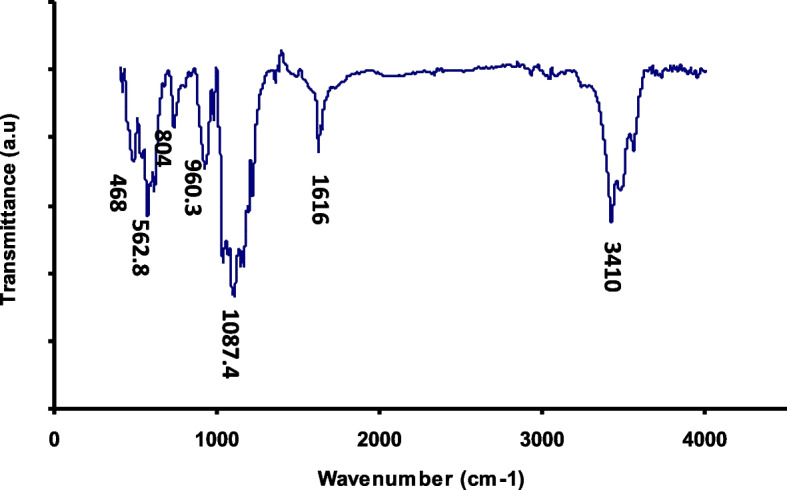
Fourier Transform Infrared (FTIR) spectrum of nBG

Fourier transform infrared spectroscopy (FTIR) was used to confirm the formation of nBG as well as to evaluate its purity. The FTIR spectrum presented in Fig. 3 shows a wide adsorption band in 3410 cm^− 1^, indicating the presence of surface hydroxyl groups. The adsorption bands at 468 and 1087.4 cm^− 1^ are related to the tensile and flexural vibrations of the Si-O-Si group, respectively. The peaks at 562.8, 804 and 960.3 cm^− 1^ are related to the flexural and tensile vibrations of the P-O group. In addition, the adsorption peak at 1616 cm^− 1^ belongs to the carbonate functional group [21].

### Microhardness evaluation

**Fig. 4:**
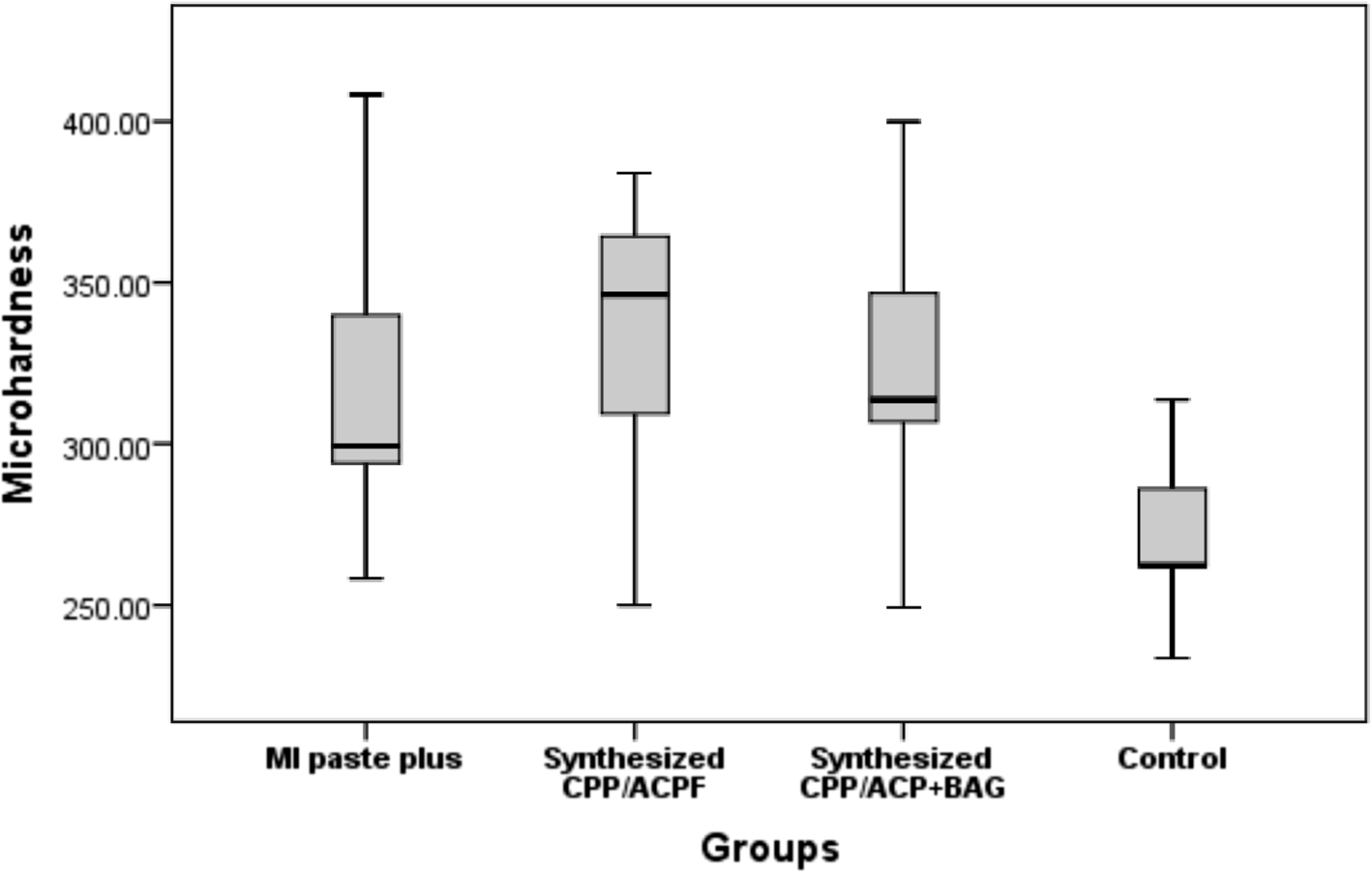
The microhardness mean ± standard deviation values of the studied groups

The microhardness mean ± standard deviations of studied groups are as follows: G1: 336 ± 35; G2: 324 ± 38; G3: 313 ± 36 and G4: 269 ± 25 (Fig. 4). The microhardness of all groups were significantly higher than the control group (*p* ≥ 0.05), however, there was no statistically significant difference between experimental groups (*p* > 0.05) (Table 1).

**Table 1:** Pair-wise comparison of the microhardness of the studied groups

Groups		Mean ± SE	*P* Value^c^
MI paste plus	Synthesized CPP-ACP@F^a^	-22 ± 12	0.262
MI paste plus	Synthesized CPP-ACP@nBG^b^	-10 ± 12	0.802
MI paste plus	control	44 ± 12	0.003
Synthesized CPP-ACP@F	Synthesized CPP-ACP@nBG	11 ± 12	0.783
Synthesized CPP-ACP@F	Control	66 ± 12	0.001
Synthesized CPP-ACP@nBG	Control	55 ± 12	0.001

^a^Casein Phosphopeptide Amoprphous Calicium phosphaste containing fluoride

^b^Casein Phosphopeptide Amoprphous Calicium phosphaste containing nano bioactive glass

^c^(Tukey post Hoc test)

**Fig. 5:**
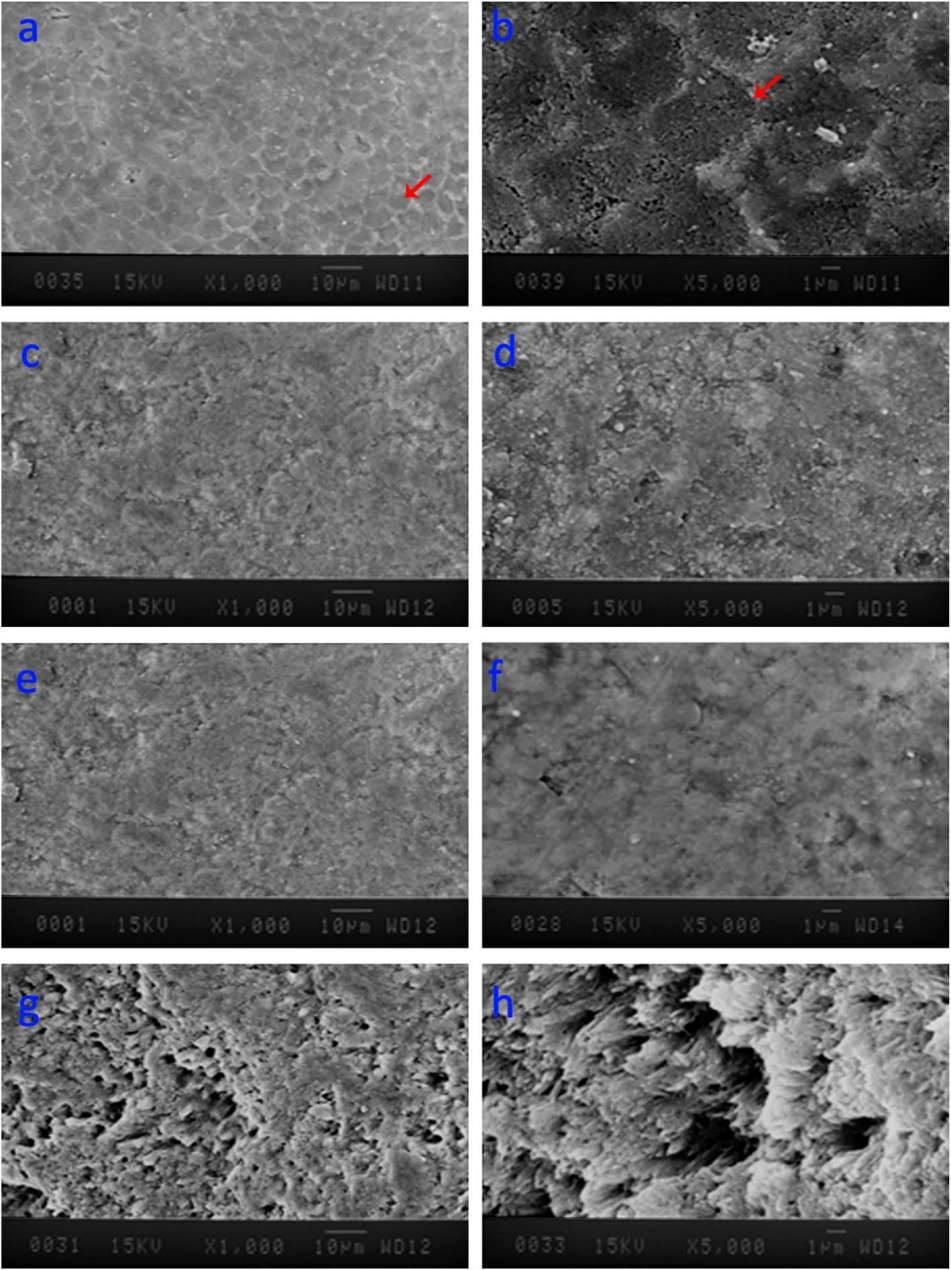
SEM (Hitachi S-450, 20 kV, Japan) images of the enamel surfaces studied. **a **Control group with ×1000 magnification. In this figure, rough and porous surface of demineralized enamel is evident; **b **Control group with ×5000 magnification depicting the honeycomb pattern in demineralized enamel surface; **c **MI-paste plus with ×1000 magnification. In this figure, homogenous deposition of hydroxyapatite on enamel surface is evident; **d **MI-paste plus group with ×5000 magnification; **e** Synthesized CPP-ACP@F with ×1000 magnification. Similar to the MI-paste plus group, homogenous deposition of hydroxyapatite on enamel surface is evident; **f **Synthesized CPP-ACP@F with ×5000 magnification, **g **Synthesized CPP-ACP@nBG with ×1000 magnification. In this figure, amorphous deposition of hydroxyapatite crystals dissimilar to those of the CPP-ACFP is evident: **h **Synthesized CPP-ACP@nBG with ×5000 magnification, note that the honeycomb pattern cannot be observed

The results of surface morphology assessment by SEM (Hitachi S-450, 20 kV, Japan) with x1000 and x5000 magnifications are presented in Fig. 5. Honeycomb view can be seen on the surface of the control group due to enamel surface demineralization during the demineralization process (Fig. 5a and b). In the CPP-ACP@F group, the porous structure of enamel prisms is covered by a uniform layer (Fig. 5e and f). Similar view is seen in the commercial CPP-ACP@F group (MI paste plus) (Fig. 5c and d). In the CPP-ACP containing nBG group (CPP-ACP@nBG), the deposition of hydroxyapatite crystals was amorphous and structurally different from the hydroxyapatite layer formed in the CPP-ACP@F group. As shown in Fig. 5 g and 5 h, the porous structure of the enamel surface has been completely covered by hydroxyapatite clusters and the honeycomb view of the enamel prisms was not visible.

## Discussion

In this study, a modified sol-gel method was used for syhntesis of nBG. X-Ray diffraction pattern (XRD) of synthesized nBG are consistent with the Larnite crystalline mode in accordance with standard card JCPDS # 33–0302 which is in agreement with the results of Federman et al. [21] and Solati et al. [15]. FTIR spectra of the synthesized nBG shows the main adsorption bands related to nBG as hydroxyl groups, Si-O-Si group, P-O group and carbonate functional group wich is in agreement with literature [15, 21]. The morphology, size distribution and shape of nBG particles wich evaluated by TEM shows its spherical nature with sized of less than 100 nm and partial agglomeration, wich is in agreement with the results of Federman et al. [21], Nikpour et al. [22] and Solati et al. [15].

The overall purpose of this study was to evaluate the effect of casein phosphopeptide-amorphous calcium phosphate containing nBG (CPP-ACP@nBG) and compare its effect with synthetic and commercial CPP-ACP-containing fluoride (CPP-ACP@F) on the enamel remineralization. The pH cycling method was used as an accelerated cariogenic condition to simulate high risk oral conditions to dental caries [18]. The results of this study showed a statistically significant difference between the study groups. The mean microhardness of the treated groups was significantly higher compared to the control group, however, the difference between mean microhardness in other groups were not significant. In SEM analysis, honeycomb pattern was evident only in the control group and in other groups the surface of enamel prisms was covered by a layer of hydroxyapatite crystals and the deposition pattern of the crystals in the CPP-ACP group containing nano bioglass was different.

Knoop and Vickers are common types of microhardness tests which are used for dental purposes. However, the Vickers test is more suitable for microhardness evaluation of tooth surface and can cause less surface bending [5]. In this study, the Vickers microhardness test was used to quantitatively evaluate the degree of enamel surface remineralization. Based on the results of the present study, the microhardness of commercial CPP-ACP@F (MI paste plus) group compared to the control group was higher which may be due to the deposition of calcium and phosphate ions on the enamel surface by CPP-ACP@F. Babu et al. found that CPP-ACP@F significantly increased enamel microhardness compared to the control group [5]. Balakrishan et al. indicated that during the enamel remineralization by CPP-ACP, microhardness significantly increased after 30 days [23]. The results of these studies are consistent with our results. As shown in Table 1, the mean microhardness of CPP-ACP@nBG was significantly higher than the control group which could be due to the synergistic effect of CPP-ACP and nBG as rich sources of calcium and phosphate ions on the tooth surface. Nano bioactive glass is composed of sodium-calcium-amorphous phosphate, and in contact with saliva its sodium ions react with hydrogen ions in saliva to release the calcium and phosphate from nBG. Following the release of sodium ions, a transient increase in pH is observed, which contributes to the deposition of calcium and phosphate, and following this, the hydroxyapatite layer forms [12]. Hyung et al. demonstrated that orthodontic bondings containing bioactive glass improved the microhardness of demineralized enamel [24]. Milly et al. reported that bioactive glass can significantly increase the microhardness of demineralized enamel [25]. Also, the microhardness of CPP-ACP@nBG was not significantly different from the synthetic and commercial CPP-ACP@F group. It is noteworthy that in this study, fluoride and nBG in combination with CPP-ACP were investigated and combination of CPP-ACP and nBG both ultimately caused hydroxyapatite deposition. The combination of CPP-ACP with fluoride results in the formation of CPP-stabilized amorphous calcium fluoride phosphate, leading more access to fluoride, calcium, and phosphate ions and the formation of fluorohydroxyapatite [2].

Palaniswamy et al. compared the effect of CPP-ACP and bioactive glass on demineralized enamel remineralization. They showed that both compounds had equally positive effects on the enamel remineralization over 15 days, however, bioactive glass was more effective in less than 10 days [26]. Soares et al. reported that both CPP-ACP@F and bioactive glass were effective in enamel remineralization [2]. The results of these studies contradict the results obtained by Bakry et al. which found that bioactive glass increased microhardness significantly more than fluoride [27].

Narayana et al. showed that CPP-ACP and bioactive glass increased the calcium and phosphate content, while CPP-ACP@F increased the calcium, phosphate, and fluoride content [28]. It has been reported that bioactive glass and fluoride reduce the depth of a demineralized lesion and fluoride-containing toothpaste has a tendency to replace the lost demineralized tissue of the lesion with remineralized material [29].

SEM assessment is a common method for preparing the topographic images and observing the occurred changes at enamel surface [28]. In this study, SEM microscopy was used to evaluate the quality of enamel surface remineralization. In the samples of synthetic and commercial CPP-ACP@F groups, hydroxyapatite crystals were uniformly deposited on the enamel prisms and the honeycomb view of enamel prisms was not visible, while the honeycomb view in enamel prisms was clearly seen. In this study, SEM assessment of groups confirmed the microhardness data. Jayarajan et al. showed that in the SEM images of remineralized enamel by CPP-ACP@F, enamel rods were not visible; however, mineral deposits were abundantly observed [3]. Also, in a study by Narayana et al. CPP-ACP@F remineralized samples showed uniform mineral deposition on the enamel surface [28]. In the present study, SEM micrographs of CPP-ACP@nBG group showed that amorphous hydroxyapatite crystals covered the porous surface of the enamel rods. Milly et al. found that in bioactive glass remineralized samples, minerals precipitated on the surface of porous enamel prisms in the form of plate-like and cubic structures [25]. Bakry et al. showed that remineralizing enamel with bioactive glass caused the deposition of crystalline structures on the entire enamel surface [27].

Rajendran et al. used SEM equipped with energy dispersing x ray analysis (EDAX) and showed that bioactive glass significantly increases the mineral content of the demineralized enamel [30]. As in vitro conditions cannot simulate clinical conditions accurately, incorporation of bioactive glass nanoparticles instead of fluoride in CPP-ACP paste needs further clinical evaluations. Under the limitations of this study, the obtained results showed that CPP-ACP@F and CPP-ACP@nBG remineralize the structure of demineralized enamel.

One of the limitations of this study can be defined as the application of pH-cycling prior to treatment. We recommend that future studies apply pH-cycling following the treatment in order to better simulate the clinical oral environment.

## Conclusion

In conclusion, the results of this study showed that CPP-ACP containing fluoride (CPP-ACP@F) and CPP-ACP containing nBG (CPP-ACP@nBG) remineralize the surface of the demineralized enamel. However, CPP-ACP@nBG remineralized teeth had higher microhardness than synthetic and commercial CPP-ACP@F remineralized one.

## Data Availability

All the data generated or analyzed during this study are included in this article.
